# Non-ketotic hyperglycaemia-induced hemichorea-hemiballism may represent glioma-like pattern on multimodal magnetic resonance imaging: can ^1^H spectroscopy help in the differentiation?

**DOI:** 10.1093/bjrcr/uaae043

**Published:** 2024-11-20

**Authors:** Yu Lin, Xiaoxiao Zhang, Xin Yue, Jinan Wang

**Affiliations:** Department of Radiology, Zhongshan Hospital Affiliated to Xiamen University, School of Medicine, Xiamen University, Xiamen, Fujian 361004, China; Department of Radiology, the First Affiliated Hospital of Fujian Medical University, Fuzhou 350004, Fujian, China; Department of Radiology, Zhongshan Hospital Affiliated to Xiamen University, School of Medicine, Xiamen University, Xiamen, Fujian 361004, China; Department of Radiology, Zhongshan Hospital Affiliated to Xiamen University, School of Medicine, Xiamen University, Xiamen, Fujian 361004, China; Department of Radiology, Zhongshan Hospital Affiliated to Xiamen University, School of Medicine, Xiamen University, Xiamen, Fujian 361004, China

**Keywords:** non-ketotic hyperglycaemia, hemichorea-hemiballismus, diabetes, glioma, magnetic resonance imaging, magnetic resonance spectroscopy

## Abstract

Non-ketotic hyperglycaemia (NKH)-induced hemichorea-hemiballismus (HC-HB) is an infrequent reversible condition observed in individuals with poorly controlled diabetes. In this report, we present a case of NKH-induced HC-HB exhibiting distinctive morphological and functional alterations on conventional magnetic resonance imaging (MRI), diffusion-weighted imaging (DWI), and ^1^H magnetic resonance spectroscopy (MRS), followed by subsequent monitoring. A 70-year-old male with a 20-year history of diabetes presented with severe unilateral involuntary movement. Computer tomography revealed heightened attenuation in the left putamen and caudate nucleus. The conventional MRI revealed the presence of focal T2-hyperintensity, noticeable mass effect, and ring-like enhancement, which are indicative of glioma. Additionally, the DWI showed unrestricted diffusion of water molecules within the lesion. MRS analysis further demonstrated significantly elevated lactate (Lac) and lipids (Lip), minimal increased choline (Cho), basically stable creatine (Cr), and modest decreased *N*-acetylaspartate (NAA) levels (which remained larger than both Cho and Cr peaks), leading to a diagnosis of NKH-induced HC-HB. This report emphasizes the significance of acknowledging that NKH-induced HC-HB can manifest with imaging features that bear resemblance to those of glioma. The presence of a slightly elevated Cho/NAA ratio alongside a notable increase in Lac/Lip peak on MRS may aid in ruling out neoplastic conditions.

## Clinical presentation

A 70-year-old male with a 20-year history of diabetes presented with severe involuntary movement of the right lower extremity for 2 days. The patient did not regularly take hypoglycaemic drugs and monitor blood glucose. His baseline non-fasting plasma glucose was 9.08 mmol/L, serum osmolality was 307.10 mOsm/L, and urinary ketone was negative.

## Imaging findings

**Figure 1.**
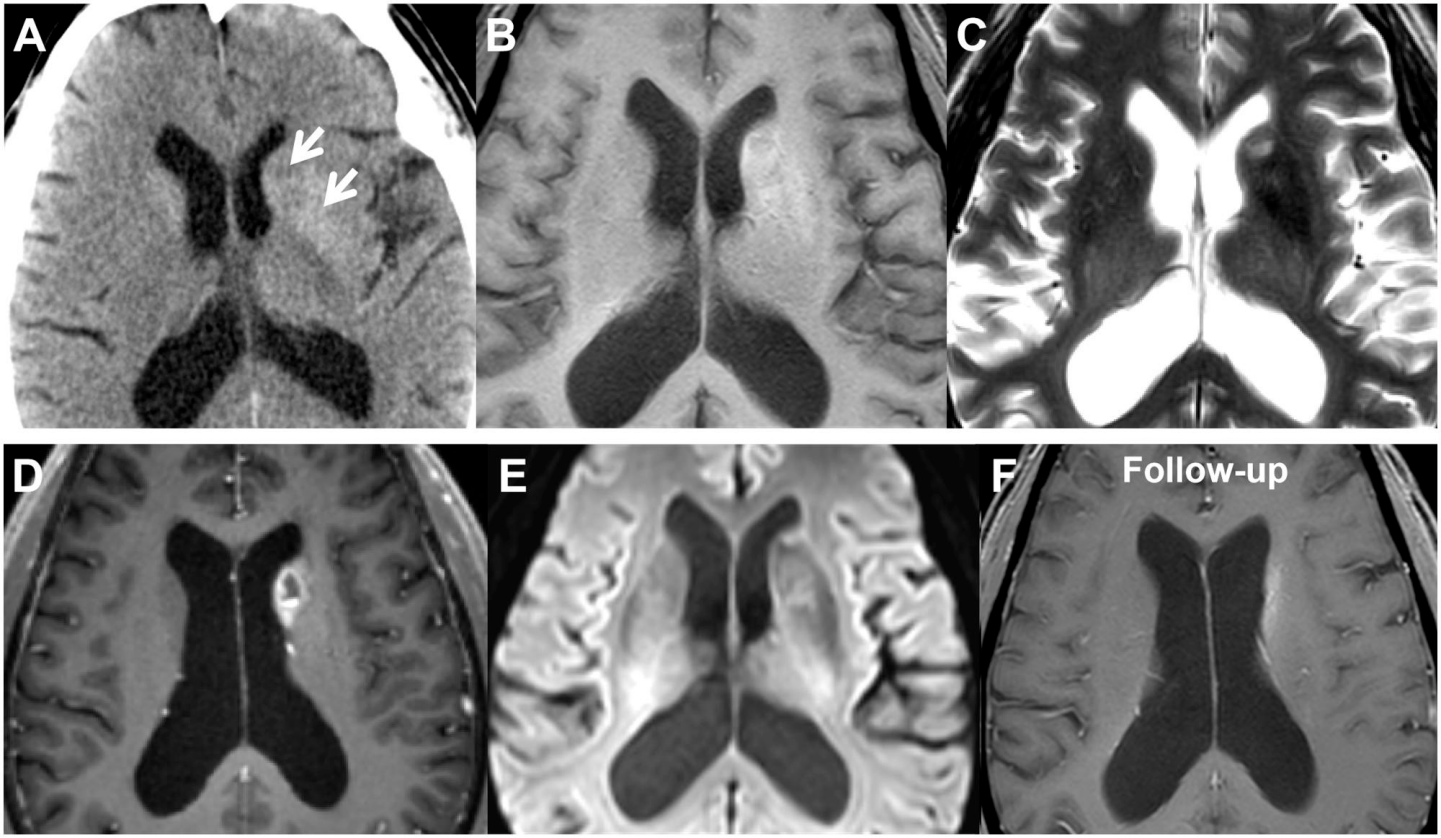
A 70-year-old male with non-ketotic hyperglycaemia-induced hemichorea-hemiballismus. Computed tomography (CT) scans demonstrated hyperdense lesions in the left putamen and caudate nucleus (A), which corresponded to hyperintense signals observed on T1-weighted magnetic resonance imaging (MRI) (B). The T2-weighted MRI (C) indicates an irregular hyperintense lesion within the caudate nucleus, accompanied by a moderate mass effect. On contrast-enhanced T1-weighted imaging (D), the lesion exhibits ring-like enhancement, while the diffusion-weighted imaging (E) shows the lesion as iso-intense or hypo-intense. A follow-up contrast-enhanced T1-weighted MRI (F) scan conducted 3 months later demonstrated a significant resolution of the previously observed lesion.

Brain computer tomography (CT) scan showed increased attenuation in the left putamen and caudate nucleus. Further magnetic resonance imaging (MRI) indicated the corresponding areas with heterogeneous hyperintensity on the T1-weighted image (T1WI). T2-weighted image and fluid-attenuated inversion recovery sequence suggested an irregular hyperintensity lesion in the caudate nucleus with a distinct mass effect (indicated by the effacement of the anterior horn of the lateral ventricle). Interestingly, the focal lesion revealed evident ring-like enhancement on contrast-enhanced T1WI, which suggested the imaging findings of glioma (Figure 1).

**Figure 2.**
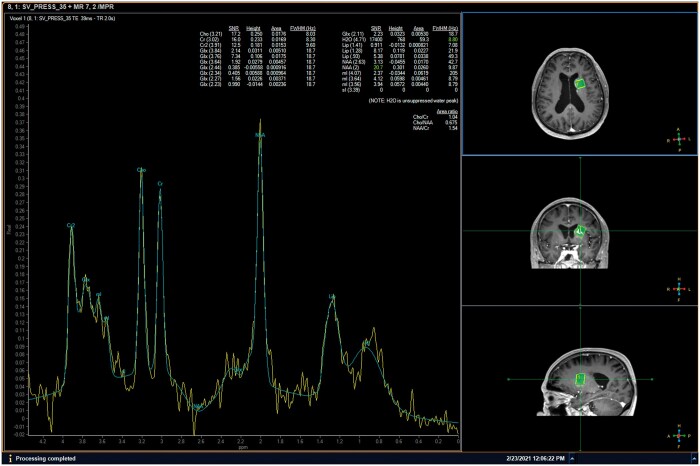
Magnetic resonance spectroscopy reveals a significantly elevated lactate peak and a mildly increased choline peak, while the *N*-acetylaspartate peak remains essentially normal.

As an advanced MRI technology, diffusion-weighted imaging (DWI) showed the lesion with iso-intensity or hypo-intensity (indicating unrestricted diffusion of water molecules within the lesion). In addition, single-voxel short echo time ^1^H magnetic resonance spectroscopy (MRS) further illustrated markedly increased lactate (Lac) and lipid (Lip) levels, slightly elevated choline (Cho) level, slightly decreased *N*-acetylaspartate (NAA) level, and basically stable creatine (Cr) level. The NAA peak exhibited a greater intensity than both the Cho peak and the Cr peak. The Cho/NAA ratio (0.675), Cho/Cr ratio (1.04), NAA/Cr ratio (1.54), and (Lac + Lip)/Cr ratio (1.34) were illustrated, respectively (Figure 2). Based on the patient’s clinical history and brain MRI results, he met the diagnostic criteria for Non-ketotic hyperglycaemia (NKH)-induced hemichorea-hemiballismus (HC-HB).

## Treatment, outcome, and follow-up

The patient’s symptoms gradually improved over days after hypoglycaemic and antiepileptic treatment. After 3 months, the patient’s clinical symptoms had essentially resolved. Follow-up MRI showed an apparent resolution of the glioma-like abnormalities in the caudate nucleus, while a patchy T1-hyper-intense area still existed in the basal ganglia region (Figure 1).

## Discussion

Diabetes mellitus is associated with pathological changes in the central nervous system that lead to impairments in neurobiology, cognition, and behaviour. NKH-induced HC-HB is a rare reversible condition of poorly controlled type 2 diabetics.^1^

Though the overall prognosis of NKH-induced HC-HB is excellent and the radiological abnormalities are generally reversible, timely neuro-imaging and accurate diagnosis are vital for such patients with acute involuntary symptoms to rule out stroke, infection, or even tumour.^2^ Typical imaging findings are increased attenuation within unilateral basal ganglia on CT and corresponding increased signal intensity on T1WI of MRI.^3^^,^^4^ In the previous literature, functional MRI techniques, including DWI and ^1^H MRS, were rarely used in the diagnosis and follow-up of NKH-induced HC-HB. In the current report, we describe a case of NKH-induced HC-HB with distinctive morphological and functional changes on conventional MRI, DWI, and ^1^H MRS.

Bedwell first described HC-HB in 1960 as an unusual movement disorder induced by poorly controlled diabetes. NKH-induced HC-HB were sporadically reported in cases with east Asian origin, which suggested hyperglycaemia as a rare cause (only 1%) of chorea with a possible genetic disposition.^1^^,^^3–5^

**Figure 3.**
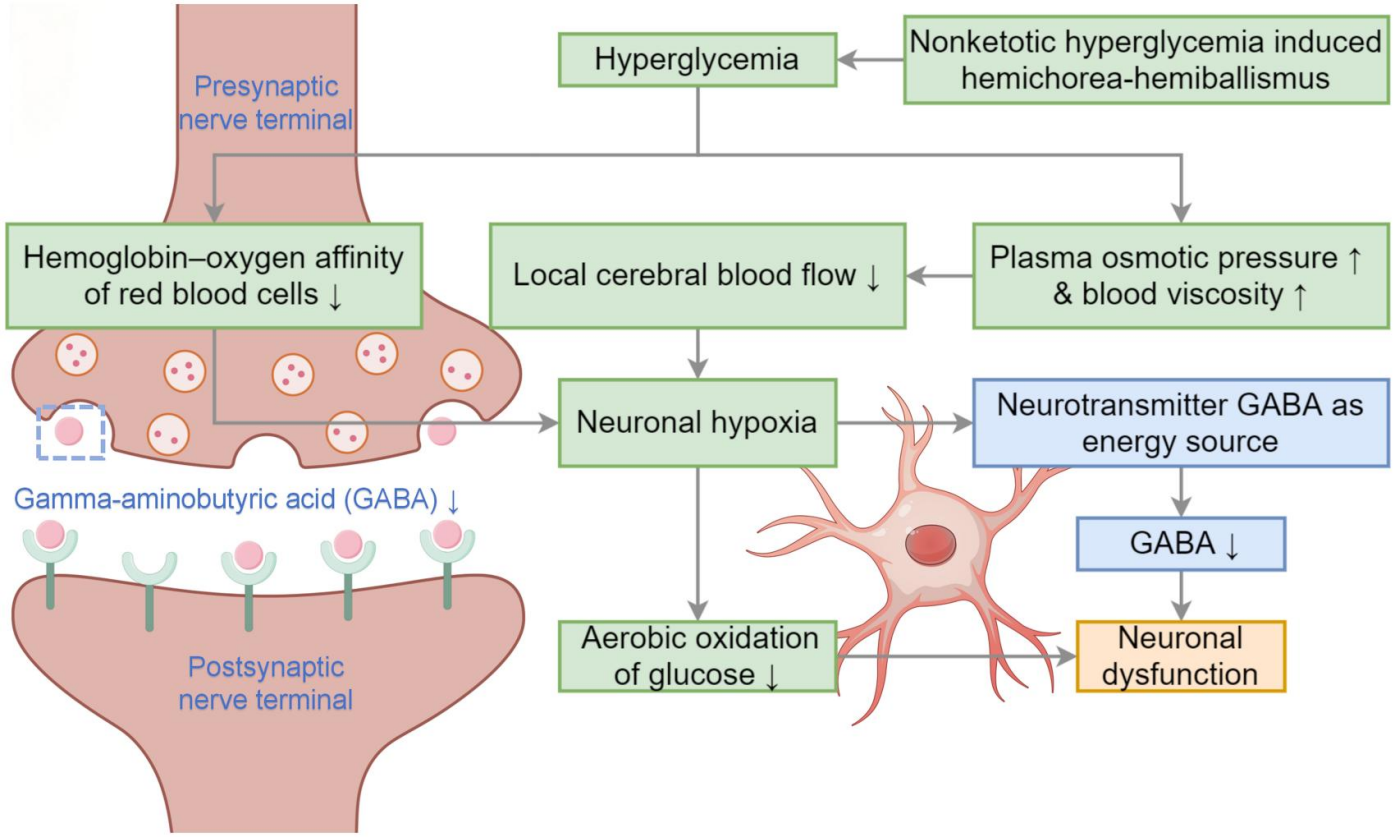
Depiction of the primary mechanism underlying non-ketotic hyperglycaemia-induced hemichorea-hemiballismus.

Although the precise mechanism of NKH-induced HC-HB remains controversial, the mainstream theory holds that hyperglycaemia causes the inhibitory neurotransmitter gamma-aminobutyric acid (GABA) to be consumed as an alternative energy source in the affected parenchyma (Figure 3).^1^^,^^5^ Consequently, reducing GABA signalling in the thalamus could lead to increased excitatory thalamocortical drive and secondary involuntary movements on the contralateral side.^5^

**Figure 4.**
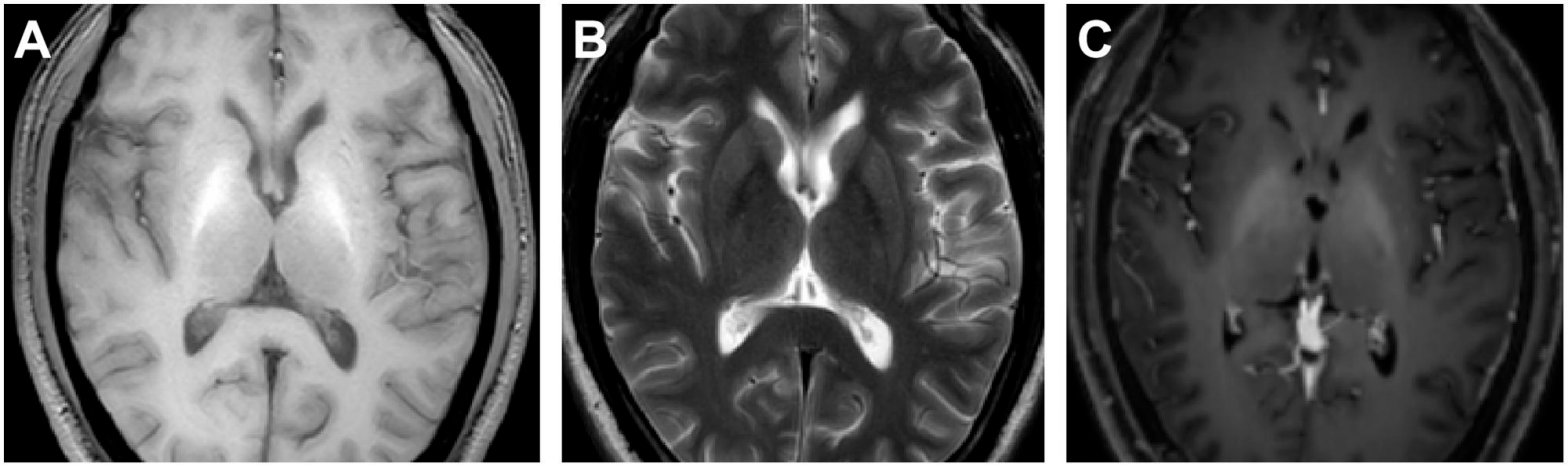
A 58-year-old male with hepatic encephalopathy. The patient presents with symmetrical abnormal signal regions in the bilateral globus pallidus. These regions demonstrate high signal intensity on T1-weighted magnetic resonance imaging (MRI) (A) and slightly low signal intensity on the corresponding T2-weighted MRI (B). Post-contrast T1-weighted MRI (C) reveals no significant enhancement.

The cause-and-effect relationship of NKH-induced HC-HB between hyperglycaemia state and imaging patterns has not yet been fully proved. The T1-hyperintensity was initially thought to be due to focal haemorrhage, demyelination, or calcification after “ischemic events,” but currently attributed to the presence of gemistocytes (special reactive astrocytes) that could accumulate manganese (causing T1 shortening effect) after chronic ischaemia.^1–4^ Other common scenarios of T1 hyperintensities attributable to manganese deposition include hepatic encephalopathy and long-term hyperalimentation, both of which symmetrically affect the bilateral basal ganglia, particularly the globus pallidus (Figure 4). This pattern can be distinguished from the unilateral onset pattern of NKH-induced HC-HB.^6^

As an unexpected discovery, the glioma-like appearance in the first case was mainly ascribed to the obvious enhancement on T1WI after injection of contrast agent. We assumed that the abnormal contrast enhancement may be due to high-glucose-induced inflammatory responses and increased blood–brain barrier permeabilities under hyperglycaemia conditions.^2^^,^^5^ It should also be noted that DWI revealed no evidence of cytotoxic oedema or active cell proliferation (presented as restricted diffusion in patients with acute cerebral infarction or high-grade glioma) in the corresponding area.

**Figure 5.**
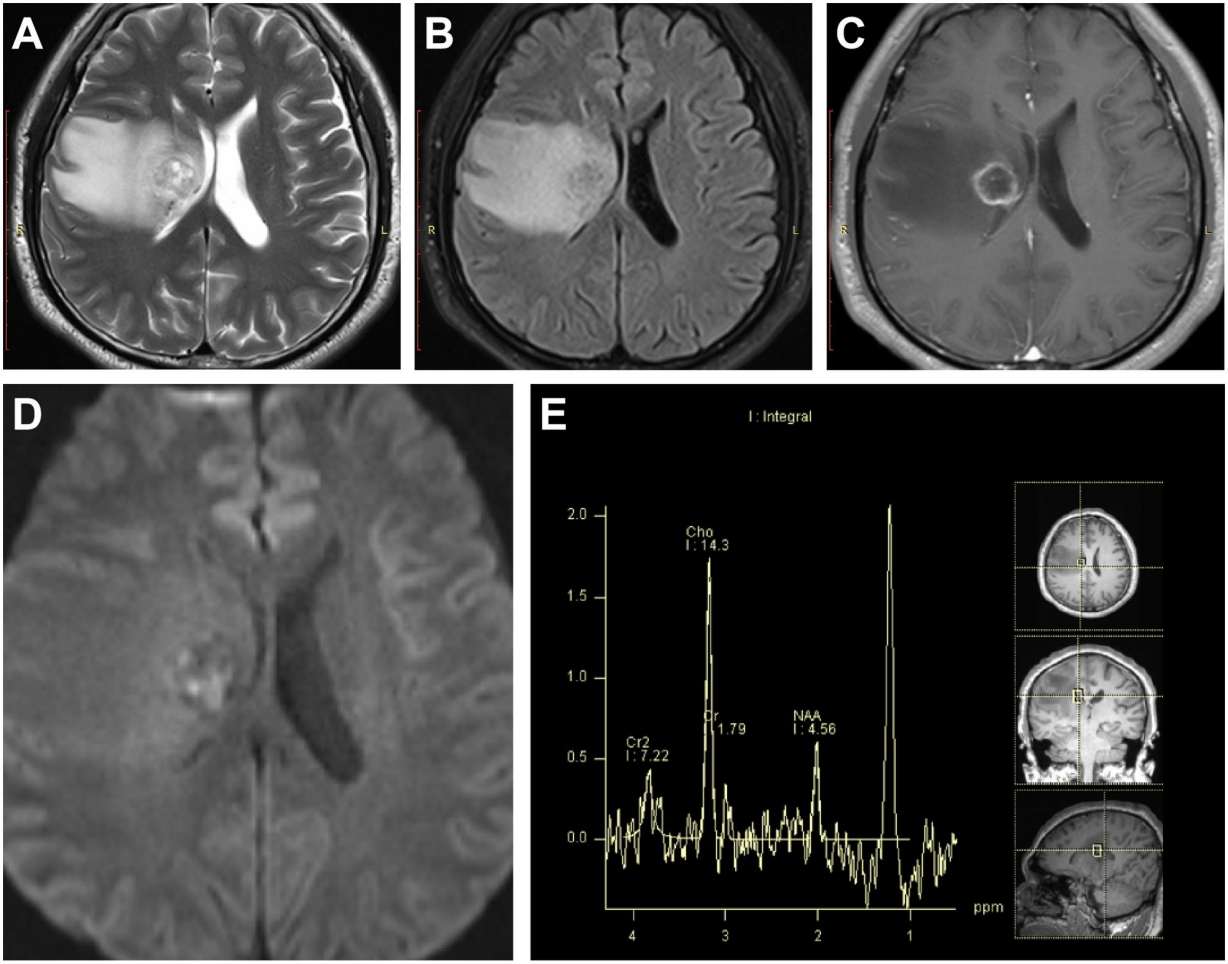
A 50-year-old male with IDH-wildtype glioblastoma. T2-weighted magnetic resonance imaging (MRI) (A) and fluid-attenuated inversion recovery imaging (B) demonstrate a heterogeneous region of high-signal intensity adjacent to the right lateral ventricle. Pronounced peritumoral brain oedema is evident. Following contrast enhancement (C), the lesion displays a ring-like enhancement pattern. Diffusion-weighted MRI (D) reveals areas of partial high signal intensity. Magnetic resonance spectroscopy (E) indicates a marked elevation in choline and lactate peaks, accompanied by a significant reduction in the *N*-acetylaspartate peak.

MRS is a non-invasive method for identifying specific metabolic markers of seizure-induced neuronal damage, which has not been systematically reported in patients with NKH-induced HC-HB.^7^ Excessive activation of various molecular pathways could lead to loss of balance between anaerobic glycolysis and oxidative metabolism (which produces large quantities of Lac) in such cases. Thus, the *in-situ* Lac level illustrated by MRS could be applied in assessing possible “ischemic damage” of NKH-induced HC-HB.^1^^,^^2^^,^^5^^,^^7^ Besides, the quantitative parameter Cho/NAA ratio of MRS is widely used in differentiating brain tumours and non-tumour diseases. In the vast majority of gliomas (especially glioblastoma), the Cho peak markedly increased (suggesting increased cell membrane synthesis), and the NAA peak markedly decreased (suggesting neuronal damage) (Figure 5). By contrast, a relatively low Cho/NAA ratio (<1) suggested the diagnosis of a metabolic/reversible disease as revealed by our case.

Moreover, brain tumours other than gliomas, such as lymphomas and metastatic tumours, can exhibit a similarly pronounced increase in the Cho peak and a decrease in the NAA peak compared to those observed in NKH-induced HC-HB, typically resulting in an inversion of the Hunter angle. Additionally, these tumours may lead to elevated Lac or Lip peaks due to processes such as cystic degeneration, necrosis, or anaerobic metabolism.^8^ Furthermore, infectious diseases impacting the central nervous system seldom involve the unilateral basal ganglia in isolation. The MRI characteristics associated with these diseases demonstrate significant variability and are often non-specific, contingent upon the particular pathogen involved. The detection of Lac, Lip, and amino acid peaks through MRS suggests the potential concurrent presence of intracranial infection and abscess formation.^9^

In conclusion, a combination of long-term uncontrolled hyperglycaemia, unilateral involuntary movements, and contralateral basal ganglia abnormalities may suggest the diagnosis of NKH-induced HC-HB. In addition to the classic T1-hyperintensity characteristic, this report emphasizes the reversible focal neurological disorder may exhibit a unique appearance mimicking glioma. A slightly elevated Cho/NAA ratio with a marked increased Lac peak on MRS may help to exclude neoplastic diseases.

## Learning points

- Imaging mimicry in non-ketotic hyperglycaemia-induced hemichorea-hemiballism (NKH-induced HC-HB): NKH-induced HC-HB can present with radiological features reminiscent of glioma, such as T2-hyperintensity, mass effect, and ring-like enhancement on MRI. This highlights the importance of considering NKH-induced HC-HB as a differential diagnosis when glioma-like imaging patterns are encountered, especially in patients with uncontrolled diabetes.
- Role of diffusion-weighted imaging (DWI) and ^1^H magnetic resonance spectroscopy (MRS) in diagnosis: The use of advanced MRI techniques like DWI and ^1^H MRS can provide valuable functional information in differentiating NKH-HC-HB from neoplastic conditions. Elevated lactate and lipids with slightly increased choline and decreased *N*-acetylaspartate on MRS, along with unrestricted diffusion on DWI, can support the diagnosis of NKH-HC-HB.
- Clinical and radiological reversibility of NKH-induced HC-HB: Although NKH-induced HC-HB is a rare but reversible condition, timely diagnosis and management are crucial to prevent misdiagnosis and unnecessary interventions. Close monitoring of blood glucose levels, hypoglycaemic therapy, and anti-epileptic treatment can lead to symptom resolution and radiological normalization, underscoring the need for a multidisciplinary approach in managing such patients.

## Data Availability

Because this is a case report, there are no associated research data to be shared.
